# Impact of distress screening algorithm for psycho-oncological needs in neurosurgical patients

**DOI:** 10.18632/oncotarget.25833

**Published:** 2018-08-03

**Authors:** Marion Rapp, Stephanie Schipmann, Kira Hoffmann, Rainer Wiewrodt, Hans-Jakob Steiger, Marcel Kamp, Walter Stummer, Dorothee Wiewrodt, Michael Sabel

**Affiliations:** ^1^ Department of Neurosurgery, Heinrich-Heine-University, Duesseldorf, Germany; ^2^ Department of Neurosurgery, University Hospital Muenster, Muenster, Germany; ^3^ Pulmonary Division, Dpt. of Medicine A, University Hospital Muenster, Muenster, Germany

**Keywords:** psychooncology, distress, brain surgery, screening, algorithm

## Abstract

**Background:**

Cerebral tumors are associated with high rates of anxiety, depression and reduced health related quality of life. Nevertheless psychooncological screening instruments are neither implemented nor well defined in the daily routine of neurosurgical departments. Therefore, we tried (1) to identify a suitable screening algorithm for neurosurgical patients, (2) to define clinical risk factors for increased distress and (3) to analyze the optimal screening time point.

**Results:**

Between October 2013 and January 2015 472 elective neurosurgical in-patients (median age 55.85 years) of the neurosurgical departments of the University Hospitals Duesseldorf and Muenster were prospectively included into this study. Regarding their diagnosis 244 (51.7%) patients were identified with malignant lesions and 228 (48.3%) patients with benign lesions. Increased distress was diagnosed in 63.1% of all patients via DT, in 13.6% via HADS and 27.8% via PO-Bado. Combining the cut-off criteria with the problem list increased sensitivity (90%) and specificity (70%) of the DT assessment. Regarding risk factors pre-existing psychiatric disorders, ataractic medication and a decreased clinical performance status were associated with increased distress.

**Patients and methods:**

Patients with diagnosis of an intracranial lesion with elective surgical indication were screened for psychological distress via three assessment-instruments the Hospital Anxiety and Depression Scale (HADS), the Distress Thermometer (DT), and the Basic Documentation for Psycho-Oncology (PO-Bado). Screening results were correlated with clinical and demographic data.

**Conclusion:**

Postoperative distress screening for neurosurgical patients is important independent from the neurosurgical diagnosis. The DT represents a suitable, non time-consuming instrument for daily routine in a neurosurgical department.

## INTRODUCTION

For most people, the brain is more than just the control center of the body, it represents identity, personality and soul. As a consequence, the diagnosis of an intracerebral process triggers great fears and anger. How to cope with a “ticking time bomb in my brain” or a “sword of Damocles hanging over my head”? Patients experience sadness; they feel ashamed of their diagnosis, appearance, or resulting deficits and withdraw more and more from social life. Up to 74% of patients report increased distress [1] with physical and emotional issues [2, 3], resulting in reduced quality of life [4, 5]. Up to 15-38% of the patients develop depressive symptoms [6, 7]. Brain cancer patients are heavily burdened, more than most other tumor patients [8, 9]. Various studies document that increased distress is not only present preoperatively but along the course of the disease [2, 3, 5], underlining the clinical impact of increased psychooncological distress in neurosurgical patients [10, 11, 12] as well as illustrating a clear correlation between elevated distress and patient compliance during further therapy [10, 13].

Despite the awareness of need for psychooncological support, there are still major shortcomings in psycho-oncological screening and the corresponding care for neurosurgical patients, especially during hospitalization. Controversial issues are (1) the most appropriate screening time-point (i.e. pre-/post-operative; in patient/outpatient), (2) the selection of the best screening instrument (i.e. HADS, DT, HIS) (3) and even about the best evaluation cut- off (i.e. in the DT from ≥4, ≥5, or ≥6) for brain tumor patients.

In 2014 the S3-guideline “Psycho-oncological diagnostics, counseling and treatment of adult cancer patients” was published by a consortium steered by the German Cancer Society. It concludes, “nationally and internationally, best evidence is available for the Hospital Anxiety and Depression Scale (HADS-D) [14]. This questionnaire is recommended as the best screening method in cancer patients.” However, the HADS is not without controversy. Apart from technical issues (calculation of cut- off value), some patients regard HADS, as it includes the question “Are you happy?” as very inappropriate and sometimes even sarcastic. Of note, the HADS was not developed specifically for cancer patients. In appreciation of these shortcomings, the S3-guidelines recommends the usage of the Distress thermometer (DT) as an alternative. The authors state, “for the psychometric quality criteria of the distress thermometer, high-quality meta-analyzes are also available internationally” [14].

Based on the existing recommendation we selected the HADS and DT as the two self-assessment tools to analyze elevated distress. In addition, the “Basic documentation for Psycho-Oncology (Po-Bado)”, a foreign-assessment instrument, was chosen as an additional screening tool for comparison. Subsequently we analysed screening results and correlated them with clinical and demographic data to identify risk factors for increased distress in neurosurgical in-patients.

In this study, 472 patients with the diagnosis of an intracranial lesion were screened for their distress, presenting - to our knowledge - the largest series of neurosurgical in-patients, comparing three different screening instruments. The aim of our study was (1) to assess the value of DT and Po-Bado in comparison with HADS as gold standard, (2) to define clinical risk factors for positive screening results and (3) to challenge the postoperative screening time- point regarding higher screening rates. Finally, taking limited time and staff resources on a neurosurgical ward into account, an easy-to-use algorithm was derived that identifies psycho-oncological overburdened patients with high specificity and sensitivity when compared with HADS.

## RESULTS

### Patients

A total of 472 patients with full data set were enrolled into the study. Baseline clinical and sociodemographic data are presented in Table 1. The mean age was 55.85 years, 221 (46.8%) patients were male and 251 (53.2%) female. 244 (51.7%) patients were diagnosed with malignant lesions (Group A), with the predominant diagnosis of glioblastoma (45.1%). Group B comprised 228 (48.3%) patients with benign lesions, with the predominant diagnosis of meningioma (47.4%). Patients in Group A suffered more often from other relevant underlying diseases, e.g. cardiovascular or renal comorbidities (p<0.001). Patients diagnosed with a benign lesion (Group B) took more often ataractics on a regular basis in comparison to patients with malignant tumors (p<0.001). There was no significant difference regarding pre-existing psychiatric disorders between both groups.

**Table 1:** Patients’ characterization- clinical and demographic data

	All patients (n= 472) n (%)	Group A (n= 244) n (%)^*^	Group B (n= 228) n (%)^*^	p-value
**Gender**				**<0.001**
**Male**	221 (46.8)	136 (55.7)	85 (37.3)	
**Female**	251 (53.2)	108 (44.3)	143 (62.7)	
**Mean age (years)**	55.85	58.18	53.35	**<0.001**
**Range**	20-90	25-85	20-90	
**Diagnosis**				**<0.001**
**Anaplastic astrocytoma**	48 (19.7)	48 (19.7)		
**Glioblastoma**	110 (45.1)	110 (45.1)		
**Metastasis**	67 (27.5)	67 (27.5)		
**Other malignant brain tumour**	19 (7.7)	19 (7.7)		
**Meningioma**	108 (47.4)		108 (47.4)	
**Pituitary adenoma**	41 (18.0)		41 (18.0)	
**Vascular lesion**	37 (16.2)		37 (16.2)	
**Other benign brain tumour**	42 (18.4)		42 (18.4)	
**Recurrence status**				**<0.001**
**Primary diagnosis**	417 (88.3)	196 (80.3)	221 (96.9)	
**First recurrence**	43 (9.1)	36 (14.8)	7 (3.1)	
**Second recurrence**	8 (1.7)	8 (3.3)	-	
**Third recurrence**	4 (0.8)	4 (1.6)	-	
**Relationship**				0.169
**Partnership**	360 (76.3)	191 (78.3)	169 (74.1)	
**Single**	104 (22.0)	49 (20.1)	55 (24.1)	
**NR**	8 (1.7)	4 (1.6)	4 (1.8)	
**Children**				0.349
**Yes**	332 (70.3)	169 (69.3)	163 (71.5)	
**No**	131 (27.8)	70 (28.7)	61 (26.8)	
**NR**	9 (1.9)	5 (2.0)	4 (1.8)	
**Employment status**				**<0.001**
**Currently employed**	87 (18.4)	30 (12.3)	57 (25.0)	
**Sick leave**	145 (30.7)	74 (30.3)	71 (31.1)	
**Retired**	182 (38.6)	116 (47.5)	66 (28.9)	
**Housekeeper**	20 (4.2)	7 (2.9)	13 (5.7)	
**Unemployed**	17 (3.6)	10 (4.1)	7 (3.1)	
**NR**	21 (4.4)	7 (2.9)	14 (6.1)	
**Comorbidities**				**<0.001**
**Yes**	128 (27.1)	83 (34.0)	45 (19.7)	
**None**	304 (64.4)	134 (54.9)	170 (74.6)	
**NR**	40 (8.5)	27 (11.1)	13 (5.7)	
**Pre-existing psychiatric disorders**				0.350
**Yes**	90 (19.1)	49 (20.1)	41 (18.0)	
**No**	371 (78.6)	191 (78.3)	180 (78.9)	
**NR**	11 (2.3)	4 (1.6)	7 (3.1)	
**Ataractics**				**<0.001**
**Yes**	117 (24.8)	47 (19.3)	70 (30.7)	
**No**	325 (68.9)	190 (77.9)	135 (59.2)	
**NR**	30 (6.4)	7 (2.9)	23 (10.1)	
**ECOG Scale**				0.612
**ECOG 0**	213 (45.1)	102 (41.8)	111 (48.7)	
**ECOG 1**	150 (31.8)	81 (33.2)	69 (30.3)	
**ECOG 2**	36 (7.6)	20 (8.2)	16 (7.0)	
**ECOG 3/4**	48 (10.2)	23 (9.4)	25 (11.0)	
**NR**	25 (5.3)	18 (7.4)	7 (3.1)	

Group A = malignant lesions, Group B = benign lesions. Univariate analysis was performed in order to determine differences between both groups. NR=not reported, ^*^percent of non missing values.

### Screening

Of 472 patients, 458 (97%) completed the HADS, the DT was completed in 453 (96%) cases and PO-Bado was available in 464 (98.3%) cases. Full screening results can be adapted from Table 2.

**Table 2:** Increased distress assessed by the different screening instruments

	HADS-D ^a^ ≥11 (n=458) n (%)	p ^d^	HADS-A ^a^ ≥11 (n=458) n (%)	p ^d^	HADS-D^a^ or HADS-A ^a^ ≥11 (n=458) n (%)	p ^d^	HADS-T ^a^ ≥22 (n=458) n (%)	p ^d^	DT ^b^ ≥5 (n=453) n (%)	p ^d^	PO-Bado ^c^ Positive (n=464) n (%)	p ^d^
**All patients**	31 (6.8%)		53 (11.6%)		64 (14%)		36 (7.8%)	0.000	286 (63.1%)		129 (27.8%)	
**Group A (malignant)**	23 (9.8%)	**0.008**	28 (11.9%)	0.814	36 (15.3%)	0.394	20 (8.5%)	0.605	148 (63.5%)	0.861	71 (29.6%)	0.375
**Group B (non-malignant)**	8 (3.6%)		25 (11.2%)		28 (12.6%)		16 (7.2%)		138 (62.7%)		58 (25.9%)	
**Male**	19 (8.9%)	0.092	18 (8.4%)	**0.048**	25 (11.7%)	0.185	20 (8.5%)	0.785	122 (57.5%)	**0.021**	55 (25.5%)	0.294
**Female**	12 (4.9%)		35 (14.3%)		39 (16.0%)		16 (7.2%)		164 (68.0%)		74 (29.8%)	
**Primary diagnosis**	27 (6.7%)	0.874	47 (11.7%)	0.870	56 (13.9%)	0.896	32 (7.9%)	0.867	252 (63.2%)	0.978	114 (27.7%)	0.931
**Recurrent disease**	4 (7.3%)		6 (10.9%)		8 (14.5%)		4 (7.3%)		34 (63.0%)		15 (28.3%)	
**Age < 65**	22 (7.0%)	0.806	41 (13.0%)	0.162	48 (15.2%)	0.263	30 (9.5%)	0.051	199 (64.0%)	0.578	94 (29.8%)	0.154
**Age > 65**	9 (6.3%)		12 (8.5%)		16 (11.3%)		6 (4.2%)		87 (61.3%)		35 (23.5%)	
**Partnership**	22 (6.3%)	0.610	40 (11.5%)	0.973	48 (13.8%)	0.851	25 (7.2%)	0.400	217(62.9%)	0.924	95 (26.4%)	0.206
**No partnership**	8 (7.8%)		12 (11.7%)		15 (14.6%)		10 (9.7%)		63 (62.4%)		34 (32.7%)	
**Children**	22 (6.9%)	0.817	40 (12.5%)	0.244	47 (14.6%)	0.453	24 (7.5%)	0.707	210 (65.8%)	**0.030**	86 (25.9%)	0.182
**No children**	8 (6.3%)		11 (8.6%)		15 (11.7%)		11 (8.5%)		69 (54.8%)		42 (32.1%)	
***Labour situation***		0.067				0.845		0.461		0.553		0.197
**Employed**	5 (5.8%)		12 (14.0%)	0.871	12 (14.0%)		9 (10.5%)		48 (57.1%)		21 (24.1%)	
**Sick leave**	6 (4.2%)		16 (11.2%)		20 (14.0%)		10 (7.0%)		91 (64.5%)		39 (26.9%)	
**Retired**	14 (8.0%)		18 (10.3%)		23 (13.1%)		11 (6.3%)		110 (62.9%)		51 (28.0%)	
**Housewife**	4 (22.2%)		3 (16.7%)		4 (22.2%)		3 (16.7%)		13 (72.2%)		6 (30.0%)	
**Unemployed**	1 (6.3%)		2 (12.5%)		3 (18.8%)		1 (6.3%)		12 (75.0%)		9 (52.9%)	
***Pre-existing psychiatric disorders***		**0.012**		**<0.001**		**<0.001**		***<0.001***		**0.031**		**0.001**
**Yes**	11 (12.4%)		21 (23.6%)		24 (27.0%)		17 (19.1%)		64 (72.7%)		38 (42.2%)	
**No**	18 (5.0%)		30 (8.4%)		38 (10.6%)		17 (4.7%)		214 (60.3%)		89 (24.0%)	
***Ataractics***		0.327		0.762		0.341		0.025		0.111		**0.025**
**Yes**	9 (8.0%)		13 (11.5%)		18 (15.9%)		41 (35.0%)		78 (69.0%)		41 (35.0%)	
**No**	17 (5.4%)		33 (10.5%)		39 (12.4%)		79 (24.3%)		189 (60.6%)		79 (24.3%)	
***underlying diseases***		0.396		**0.008**		0.082		0.875		0.103		0.378
**Yes**	6 (4.9%)		22 (17.9%)		23 (18.7%)		8 (7.1%)		83 (68.6%)		39 (30.5%)	
**No**	21 (7.1%)		26 (8.8%)		36 (12.2%)		21 (6.6%)		176 (60.1%)		80 (26.3%)	
***ECOG*** ^e^		**0.019**		0.539		0.057				0.269		**<0.001**
**ECOG 0**	13 (6.3%)		21 (10.2%)		24 (11.7%)		15 (7.2%)	0.431	122 (59.8%)		39 (18.3%)	
**ECOG 1**	4 (2.7%)		15 (10.1%)		16 (10.8%)		8 (5.4%)		89 (61.0%)		40 (26.7%)	
**ECOG 2**	3 (9.1%)		6 (18.2%)		7 (21.2%)		4 (12.1%)		24 (72.7%)		19 (52.8%)	
**ECOG 3/4**	7 (15.2%)		6 (13.0%)		11 (23.9%)		5 (10.9%)		33 (71.7%)		26 (57.8%)	

Univariate analysis was performed in order to compare different subgroups regarding their presence of psychooncological distress.

^a^ Hospital Anxiety and Depression Score (D= Depression, A= Anxiety).

^b^ Distress thermometer.

^c^ Basic Documentation for Psycho-Oncology.

^d^ for comparison of screening results of the different assessment instruments and different subgroups of patients. Bold printed values indicate significance (p<0.05).

^e^ Eastern Cooperative Oncology Group performance status.

In Group A, 44 (26.5%) patients with positive screening for psychooncological distress and 4 (6.2%) negative screened patients whished further psychooncological consultation (p=0.001). In Group B, 24 (16%) and 5 (7.4%) patients, respectively, accepted the offer of further support (p=0.082).

### Hospital anxiety and depression scale (HADS)

Thirty-one (6.8%) patients were screened positive in the HADS depression score (HADS-D). Patients with malignant lesions (Group A: n=23, 9.8%) had relevant higher depression scores than patients with benign lesions (Group B, n=8, 3.6%, p= 0.008). In addition, a poorer ECOG performance status (p=0.019) and the presence of pre-existing psychiatric disorders (p=0.012) were associated with a pathological HADS-D screening result (Table 2).

Analysis of the HADS anxiety score (HADS-A) revealed more patients in total (11.6%) and among them more females with a pathological screening results (p= 0.048, Table 2), whereas there was no difference regarding the benign or malignant differentiation of the underlying lesion (p= 0.814, Table 2). In addition, pre-existing psychiatric disorders and underlying diseases significantly influenced the screening results (p<0.001; p=0.008, respectively, Table 2).

We further calculated the two combined variables, HADS-T (cut-off level ≥ 22) and “HADS-A or HADS-D” (cut-off levels ≥ 11 for both). As expected, clinical associations of distressed patients are in line with the results of HADS-A and HADS-D, respectively (Table 2).

Multivariate analysis: Of interest, multivariate analysis of HADS-A and HADS-D were very much in line with univariate results (Table 3). When calculating for HADS-T, multivariate analysis revealed a very prominent signal for a higher risk for pathological scores in patients with pre-existing psychiatric disorders (OR: 4.30, 95%CI: 1.93-9.58, p= 0.001) exclusively.

**Table 3:** Multivariate analysis revealing risk factors associated with pathological screening results in the different assessment tools

	HADS-D^a^ ≥11		HADS-A^a^ ≥11		HADS-T^a^ ≥22		DT^b^ ≥5		Emotional problems ≥ 2	
	OR^d^ (95% CI)^e^	p	OR ^d^ (95% CI)^e^	p	OR^d^ (95% CI)^e^	P	OR^d^ (95% CI)^e^	p	OR^d^ (95% CI)^e^	p
**Brain tumor entity**										
**Group A (malignant)**			-		-		-		-	
**Group B (benign)**	3.82 (1.36-10.69)	**0.005**		n.s.^f^		n.s.^f^		n.s.^f^		n.s.^f^
**Gender**										
**male (reference)**			-				-			
**female**	-	n.s.^f^	2.02 (1.01-4.06)	0.042	-	n.s.^f^	1.79 (1.13-2.74)	**0.007**	-	n.s.^f^
**Age**	-	n.s.^f^	-	n.s.^f^	-	n.s.^f^	-	n.s.^f^	0.98 (0.96-0.99)	**0.004**
**Pre-existing psychiatric disorders**										
**No (reference)**			-		-		-		-	
**Yes**	-	n.s.^f^	3.18 (1.61-6.29)	**0.001**	4.30 (1.93-9.58)	**0.001**	1.86 (1.06-3.28)	**0.027**	2.24 (1.31-3.81)	**0.003**
**ECOG**										
**ECOG 0 (reference)**								n.s.^f^		
**ECOG 1**	-	n.s.^f^	-	n.s.^f^		-	-		2.34 (1.44-3.81)	**0.001**
**ECOG 2**	-	n.s.^f^	-			-	-		2.59 (1.15-5.83)	0.022
**ECOG ¾**	3.76 (1.32-10.77)	**0.005**	-			-	-		2.82 (1.31-6.10)	**0.008**

Of note, there were not statistically significant correlations observed for PO-Bado^c^. The following variables were included in multivariate analysis: dignity of lesion (Group A: malignant vs. Group B: benign (ref), sex (female vs. male (ref)), disease status (first (ref) vs. recurrent), age, partnership (yes vs. no (ref)), children (yes vs. no (ref)), labor situation (working (ref) vs. sick leave, retired, housewife, unemployed), pre-existing psychiatric disorders (yes vs. no (ref),, regular medication with ataractics (yes vs. no (ref)), presence of underlying diseases (yes vs. no (ref)), ECOG performance status (ECOG 0 (ref), vs. EGOG 1, ECOG 2, ECOG 3/4).

^a^ Hospital Anxiety and Depression Score (D= Depression, A= Anxiety, T=Total).

^b^ Distress thermometer.

^c^ Basic Documentation for Psycho-Oncology.

^d^ Odd ratio.

^e^ 95% Confidence Interval.

^f^ not significant.

### Distress thermometer (DT)

The DT showed pathologically high scores (scoring ≥5) in 286 patients (63.1%). Factors with a higher risk for pathological screening were gender and pre-existing psychiatric disorders both in univariate and multivariate analyses (Tables 2 and 3).

All items of the problem list were used in this study. Physical problems were endorsed most often (n=403, 90%), followed by emotional problems (n=269, 60.0%), while practical (n=102, 22.7%), family (n=32, 7.1%) and spiritual (n=27, 6.1%) problems were less often present.

To understand which of the five problem groups may influence the overall DT score, regression analyses were performed. DT score was most influenced by emotional problems both in patients with malignant and benign lesions, respectively (R=0.439 and R=0.491, resp., both p< 0.001), followed by physical problems (R=0.402 and R=0.364, resp., both p< 0.001y) and practical problems (R=0.206, p=0.002; R=0.275, p< 0.001, respectively), whereas spiritual and family problems did not correlate with the DT score.

### Psycho-oncological base documentation (PO-Bado)

Screening with PO-Bado revealed the need for psychooncological intervention in 129 (27.8%) patients. We did not find any statistical significant differences between patients with malignant and benign lesions (p= 0.375). Univariate analysis revealed pre-existing psychiatric disorders (p= 0.001), regular medication with ataractics (p= 0.025) and a high ECOG performance score (p< 0.001) as being significantly associated with a pathological screening result in the PO-Bado instrument (Table 2). In multivariate analysis, there were no significant associations observed.

### Screening time-point

Because of the admission and inpatient processes in both departments patients were predominantly screened after surgery (n=444, 94.1%), with few being screened prior brain surgery (n=28, 5.9%). There was no difference in positivity rate regarding pre- or post-surgical screening, with neither screening instrument (p>0.05, all comparisons). 294 patients (62.3%) were assessed one or two days after surgery and 150 patients (31.8%) on day three or later after surgery. With all screening instruments, screening at day 3 or later revealed the highest positivity rates. However, in all cases of HADS (HADS-A, -D, -T, and -A or D) this difference did not reach statistical relevance (p≥ 0.05, all comparisons; of note, p=0.050 when using the combined variable “HADS-A ≥ 11 or HADS-D ≥ 11). In contrary, applying DT (cut-off levels ≥ 5; ≥ 6), DT problem list (emotional problems, cut-off levels ≥ 1; ≥ 2; ≥ 3), and PO-Bado, postoperative screening at day 3 or later yielded in significant higher rates of distressed patients (p< 0.05, all comparisons; data not shown).

### Sensitivity, specificity and receiver operating characteristics (ROC)

The absolute number for positive test results between HADS and DT differed widely. Positive test results for DT (score ≥ 5) were found in 63.1% of cases. In contrast, HADS-A was only positive in 11.6%, HADS-D and HADS-T were even lower (6.8% and 7.6%, respectively). Combining “HADS-A or HADS-D” increased the positivity rate up to 13.6% (Table 2). Given that HADS is the gold standard to detect clinical relevant psycho-oncological problems, screening with DT would reveal a sensitivity of approximately 93% and a specificity of approximately 40% for a positive HADS screening result, depending on which HADS-subcategory was looked at (Table 4).

**Table 4:** Receiver operating curves (ROC)

		DT^b^			Practical problems			Emotional problems			Physical problems			PO Bado^c^		
		AUC^d^	p^e^	95%CI^f^	AUC^d^	p^e^	95%CI^f^	AUC^d^	p^e^	95%CI^f^	AUC^d^	p^e^	95%CI^f^	AUC^d^	p^e^	95%CI^f^
**HADS-A**^a^	**benign**	0.714	0.000	0.621-0.807	0.633	0.033	0.505-0.762	0.908	0.000	0.860-0.956	0.746	0.000	0.645-0.839	0.765	0.000	0.657-0.872
	**malignant**	0.740	0.000	0.648-0.832	0.643	0.013	0.525-0.765	0.886	0.033	0.821-0.952	0.726	0.000	0.636-0.817	0.734	0.000	0.629-0.840
	**all**	0.728	0.000	0.663-0.794	0.640	0.001	0.552-0.727	0.897	0.000	0.856-0.938	0.737	0.000	0.672-0.802	0.746	0.000	0.674-0.824
**HADS-D**^a^	**benign**	0.825	0.002	0.730-0.919	0.779	0.008	0.599-0.959	0.901	0.000	0.814-0.989	0.738	0.022	0.619-0.858	0.824	0.002	0.685-0.962
	**malignant**	0.723	0.000	0.618-0.828	0.643	0.024	0.5214-0773	0.846	0.000	0.765-0.927	0.803	0.000	0.726-0.880	0.792	0.000	0.693-0.891
	**all**	0.747	0.000	0.664-0.830	0.677	0.001	0.569-0.785	0.854	0.000	0.790-0.919	0.772	0.000	0.706-0.839	0.801	0.000	0.720-0.882
**HADS-A^a^ or HADS-D^a^**	**benign**	0.735	0.000	0.650-0.821	0.652	0.011	0.530-0.774	0.913	0.000	0.866-0.959	0.748	0.000	0.663-0.833	0.766	0.000	0.663-0.868
	**malignant**	0.698	0.000	0.611-0.785	0.619	0.024	0.511-0.726	0.850	0.000	0.781-0.919	0.747	0.000	0.670-0.823	0.733	0.000	0.638-0.829
	**all**	0.714	0.000	0.653-0.776	0.633	0.001	0.552-0.713	0.877	0.000	0.833-0.921	0.746	0.000	0.688-0.803	0.748	0.000	0.679-0.818
**HADS-T**^a^	**benign**	0.808	0.000	0.712-0.904	0.712	0.006	0.558-0.876	0.938	0.000	0.892-0.983	0.738	0.002	0.628-0.848	0.762	0.001	0.628-0.896
	**malignant**	0.764	0.000	0.649-0.878	0.704	0.003	0.567-0.842	0.906	0.000	0.856-0.957	0.753	0.000	0.655-0.850	0.828	0.000	0.740-0.917
	**all**	0.785	0.000	0,708-0.861	0.708	0.000	0.605-0.81	0.92	0.000	0.886-0.955	0.745	0.000	0.672-0.819	0.8	0.000	0.722-0.878

ROC analyses were performed using HADS as the gold-standard against the other screening instruments. The area under the curve (AUC) reflects the overall performance of the other test instruments in discriminating between patients with and without positive screening results in HADS. An AUC between 0.7 and 0.8 reflects fair discrimination results, between 0.8 and 0.9 a good and above 0.9 an excellent discrimination.

^a^ Hospital Anxiety and Depression Score (D= Depression, A= Anxiety, T=Total).

^b^ Distress thermometer.

^c^ Basic Documentation for Psycho-Oncology.

^d^ Area under the curve.

^e^ p-value.

^f^ 95%-Confidence Interval.

Since ROC analyses revealed that the presence of emotional problems discriminated the best between distressed and non-distressed patients with benign and malignant tumors (Table 4), we combined the global DT score with results of the DT problem list.

By adding the DT problem list results, specificity of DT screening can be highly increased. DT ≥5 and presence of emotional problems revealed not only a higher specificity but resulted in an only discrete loss in sensitivity (Table 5). Reporting at least 2 emotional problems, a clinical very meaningful sensitivity and specificity with regard to HADS as gold standard was reached, independent of the global DT score (Table 5). Combining the criteria DT ≥ 5 and emotional problems ≥ 2, a concerted, clinically meaningful sensitivity-specificity balance could be achieved (sensitivity of approx. 90%, specificity of approx. 70%).

**Table 5:** Sensitivity and specificity for determination of the optimum cutoff scores of the DT and different combinations of DT and emotional problems from the DT problem list

	Sensitivity (%)				Specificity (%)			
	HADS-A^a^ ≥11	HADS-D^a^ ≥11	HADS-D^a^ or HADS-A^a^ ≥11	HADS-T^a^ ≥11	HADS-A^a^ ≥11	HADS-D^a^ ≥11	HADS-D^a^ or HADS-A^a^ ≥11	HADS-T ^a^ ≥11
**DT^b^ ≥5**	92.5	93.5	93.5	91.7	40.8	39.1	41.7	39.3
**DT^b^ ≥5 or emotional P^d^ ≥1**	100	100	100	100	24.3	23.1	25	23.4
**DT^b^ ≥5 or emotional P^d^ ≥2**	100	100	100	100	33.2	31.6	34.1	31.9
**DT^b^ ≥5 or emotional P^d^ ≥3**	98	100	98.4	100	35.2	33.7	36.2	34.1
**DT^b^ ≥5 and emotional P^d^ ≥1**	94.1	90.3	90.3	94.3	61.5	58.6	62.5	59.5
**DT**^b^ **≥5 and emotional P**^d^ **≥2**	94.1	87.1	88.7	94.3	71.9	68.2	72.9	69.3
**DT^b^ ≥5 and emotional P^d^ ≥3**	90.2	80.6	82.3	94.3	77.7	73.7	78.4	75.4
**DT^b^ ≥6**	70.6	71	66.1	75.0	58.5	57.1	58.6	58.0
**DT^b^ ≥6 or emotional P^d^ ≥1**	100	96.8	98.4	100	32.7	30.8	33.3	31.4
**DT^b^ ≥6 or emotional P^d^ ≥2**	100	96.8	98.4	100	45.3	42.9	46.4	43.6
**DT^b^ ≥6 and emotional P^d^ ≥1**	66.0	71.0	62.5	75.0	70.9	69.3	71.3	70.1
**Emotional P^d^ ≥1**	98	96.8	96.8	100	44.8	42.7	45.9	43.3
**Emotional P^d^ ≥2**	98	93.5	95.2	100	64	60.7	65.3	61.7
**Emotional P^d^ ≥3**	92.2	87.1	87.1	100	72	68.6	73.1	70.2
**PO-Bado^c^ positive**	71.2	83.3	69.8	80.0	78.6	76.9	79.8	77.2

The HADS was used as a gold-standard.

^a^ Hospital Anxiety and Depression Score (D= Depression. A= Anxiety. T=Total).

^b^ Distress thermometer.

^c^ Basic Documentation for Psycho-Oncology.

^d^ Emotional Problems.

## DISCUSSION

With this large, prospective study including patients who underwent elective brain surgery we aimed to answer several clinically very relevant questions regarding psychooncological distress. HADS and DT were selected as the two self-assessment tools in the study. In addition, the “Basic documentation for Psycho-Oncology (Po-Bado)”, an external-assessment instrument, was chosen as an additional screening tool for comparison. To our knowledge, it is the largest series of neuro-oncological in-patients screened so far in order determine an algorithm for a fast and reliable screening on a busy neurosurgical ward.

One striking observation of our study is the high variability of positive screening results among the self-screening instrument HADS (13.6%, HADS-A ≥ 11 or HADS-D ≥ 11, with various rates for depression (6.8%) and anxiety (11.6%)), the self-screening instrument DT (63.1%; DT ≥ 5) and the foreign-assessment tool PO-Bado (27.8%). As a consequence, and given the HADS as gold standard, we addressed which of the alternative screening instruments reflect HADS most appropriately. Our results for HADS appear somewhat lower than described in literature. Goebel and Mehdorn studied 150 in-patients (pre-/post-op) as well as outpatients and found in 22.7% symptoms of anxiety and 8.7% of depression [2]. In another study of the same author lower numbers were reported as well. They examined a small group of 26 patients over a 6-month period and found values of 12% in HADS-A and 4% in HADS-D within the first 3 months after the initial diagnosis [1], potentially reflecting the variability of screening results due to different screening time-point.

In the same study DT screening results were analysed as well. In this small patient collection with the cut-off DT ≥ 5, 74.4% patients demonstrated suspicious striking results [1]. Our larger study demonstrated comparable high positive rates, in line also with reports by other authors [5, 15, 16]. Due to the ongoing discussion about the “correct” cut-off value in DT in brain tumor patients with values ranges from ≥4 to ≥6 [4], we assessed sensitivity and specificity with DT ≥5 and DT≥6. As expected, applying DT≥6, sensitivity was reduced while specificity was higher compared with DT ≥5 (Table 5). Surprisingly, our percentages were far-off the reported data. A meta-analysis by Ma et al. of 42 studies with 14,808 tumor patients summarized that a cut-off of 4 is optimal [17]. The pooled sensitivity in DT was 81%, pooled specificity 72%. When the DT was compared to the value of HADS-T (total), there was a pooled sensitivity of 0.82 (95% CI 0.80-0.84) and a pooled specificity of 0.73 (95% CI 0.72-0.74). The AUC was 0.8432. In our bicentric, prospective study, the values for specificity were disappointing (i.e. DT ≥5 / HADS-T): sensitivity 91.7 %, specificity 39.3%, AUC 0.785.

As a result, we aimed for alternative DT criteria indicative for patients with high psycho-oncological burden and analyzed the problem list result in more details. In our study the majority of problems are of physical nature (≥ 1 physical problem: 90%), which can be explained by symptom leading to neurosurgery and the surgical intervention by itself. The second most common reported problem group were emotional problems (60%), defined as worries, fears, grief or loss of interest. Renovanz et al. also found similar results: physical problems (88.0%), emotional problems (70.9%) [3].

Using ROC analyses, we identified the DT problem list subgroup “emotional problems” to best reflect burdened neurosurgical patients. 23,6% patients reported three or more emotional problems, resulting in: sensitivity 100%, specificity 70.2%, AUC 0.920 (all calculations with respect to HADS-T). To our knowledge, we are the first to deduce the DT problem list subgroup “emotional problems” as an alternative self-assessment option with an at least equivalent diagnostic power to identify distressed patients.

In addition, the combination of overall DT-score and/or problem list assessment may be combined (Table 5). The application of DT *or* emotional problems resulted in a higher sensitivity but lower specificity, hence this composite score may not be convincing in clinical practice. On the contrary, when combining DT *and* emotional problems, sensitivity and specificity reach the same range than the values for Po-Bado, a resource intensive physician-based, foreign assessment tool.

Regarding PO-Bado, 27.8% of our patients had a suspicious screening result. Compared to HADS (13.6% positive), PO-Bado is more labor-intensive and time-consuming. As a result, in our view, this screening instrument is unrealistic in everyday clinical practice, even if the intensive conversation is certainly exceptional value for the patient and the physician receives a lot of additional information about the patient. Application of the self-assessment instrument DT (global score and DT problem lists) is as time efficient as HADS, but even easier to read and calculate. When comparing sensitivity and specificity of HADS (i.e. HADS-A) with PO-Bado and DT, we could show that combining DT≥5 and emotional problems ≥3 resulted in a higher sensitivity than PO-Bado (90.2% versus 71.2%, resp.) while remaining the same specificity (77.7% versus 78.6%, resp.) (Table 5). As a consequence, we favor DT and /or DT emotional problems over PO-Bado.

### Establishing a screening algorithm for routine use

**Figure 1:**
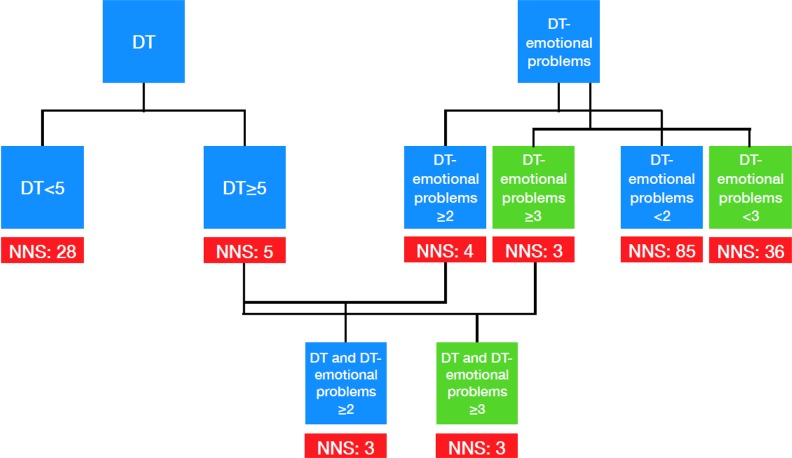
Screening algorithm for clinical practice

Based on the above-mentioned analyses we propose a screening algorithm (Figure 1). Taking only the absolute DT score into account with a cutoff of ≥ 5, the number needed to screen (NNS) would be 5, indicating that 5 patients must be screened to identify one patient with a positive HADS screening result and the need for psycho-oncological support. The number needed to screen can be reduced to 3 when combining the DT score with the DT emotional problem list (≥ 2 or ≥ 3 emotional problems). In addition, patients with ≥ 2 and ≥ 3 positive items on the emotional problem list regardless of the DT score result are likely to be screened positive in HADS as well (NNS = 4 and NNS = 3, respectively).

Therefore, the analysis of both the DT score and the results for emotional problems are clinically very meaningful. Moreover, the presence of at least one emotional problem showed a higher sensitivity and specificity compared with DT ≥5. Hence, in clinical use, both overall DT score and the DT problem list should be indispensable components of any psych oncological self-assessment.

Regardless of the selection of a certain screening tool, we identified important clinical risk factors for high psycho-oncological burden. The presence of pre-existing psychiatric disorders is the most relevant independent risk factor. Depending on the screening instrument, female gender, younger age, malignancy, and ECOG-performance status are additional independent risk factors. Another very important finding of our study was that screening may be performed after surgery at day 3 or later, yielded in significant higher rates of distressed patients compared with screening at an earlier time point when using DT and/or PO-BADO as screening tool. However, using HADS as screening measure, the screening time point.

Our study has some limitations. Validity of screening results were not independently verified by a psychiatrist or psycho-oncologist in every patient screened. Therefore, the real necessity for specific interventions is not known. Patient selection was limited, only inpatients after elective craniotomy were screened during their hospital stay. The value of psycho-oncological interventions and follow-up screening during the further course of the disease remains to be answered.

Future aims are to find out whether brain tumor-specific interventions are desired or necessary, or whether, in addition to psych education, general behavioral therapy principles for dealing with anxiety and worry are sufficient. Since, according to our own experience, especially at the beginning of the disease, many questions about the tumor, the therapy or dying of a brain tumor are the cause of the worries and fears [18], it is a great advantage for neurosurgical colleagues to continue their education as psycho-oncologists in order to reduce fears in the context of a better psych education, as the physical symptoms and fatigue should be mentioned first and foremost, an intensive sports program for brain tumor patients should be offered early.

In summary, 472 patients with diagnosis of an intracranial lesion were screened after surgery with the three tools. The first aim was to assess the value of DT and Po-Bado in comparison with HADS as gold standard. Both, overall DT score and the DT problem list, especially the emotional problem section should be indispensable components of any psycho-oncological self-assessment with a very low number needed to screen in comparison with HADS as gold standard. Second, we showed that screening at least three days after surgery yields in higher screening rates compared with screening at an earlier time point. Third, besides gender, age, and ECOG performances status, the most relevant clinical risk factor for high psycho-oncological burden was the presence of a pre-existing psychiatric disorder. In order to reliably facilitate screening for all patients on a neurosurgical ward, an easy-to-use algorithm was derived that identifies psycho-oncological overburdened patients with high specificity and sensitivity when compared with HADS.

## PATIENTS AND METHODS

### Study design and patients

Between October 2013 and January 2015 patients with diagnosis of an intracranial lesion, which were operated in the Departments of Neurosurgery of the University Hospitals Dusseldorf and Muenster, were prospectively included into this bi-center study. Patients were screened for psychological distress via two self-assessment instruments (Hospital Anxiety and Depression Scale (HADS) and Distress Thermometer (DT)) and one external assessment questionnaire (Psychooncological base documentation (PO-Bado). Intracranial lesions comprised malignant and benign brain tumors as well as vascular lesions.

Inclusion criteria were elective admission due to a neurosurgical procedure, age ≥ 18 years, and informed written consent. Patients under palliative care and patients with physical or cognitive inability to complete the screening instruments were not included into the study.

After inclusion, patients were divided into two groups according to the benign or malignant differentiation of their cerebral lesion. Group A comprised patients with malignant lesions, including gliomas, metastases and other malignant brain tumors, whereas patients in group B were diagnosed with benign lesions, like meningiomas, vestibular schwannomas, pituitary adenomas or vascular lesions (cavernous malformations). Further detailed patients’ characteristics are summarized in Table 1.

The following medical information was collected systematically: age, gender, underlying diseases (cardio-vascular, renal and pulmonary), status of disease, EGOC (Eastern Cooperative Oncology Group) performance status, neurosurgical diagnosis, social factors like relationship status, children and occupation. In addition, data regarding pre-existing psychiatric disorders and medication with ataractics were collected (Table 1).

### Screening instruments

Patients were asked to complete three screening instruments, the Hospital Anxiety and Depression Scale (HADS-A/HADS-D), the Distress Thermometer (DT), and the Basic Documentation for Psycho-Oncology (PO-Bado).

**HADS** was originally designed to assess the psychological state of physically ill patients [19]. Meanwhile it has been established as an effective screening tool for assessment of anxiety and depression in psycho-oncological practice [20–22]. The 14-item self-report questionnaire consists of 7 items used to identify anxiety (HADS-A) and 7 items for depression (HADS-D), with each item having a 4-point (0-3) Likert-type scale. Scores for each subscale range from 0- 21, higher scores indicate greater anxiety and/or depression. Two thresholds are recommended: ≥8 for greater sensitivity and ≥11 for greater specificity [23]. We used a cut-off of ≥ 11 to define a pathological HADS-A or HADS-D screening result. In addition, the total score HADS-T is defined pathological when it is above 22.

**DT** is a single-item visual analogue scale (ranging from 0 to 10 (maximal distress)), developed to rapidly screen patients for psychological distress, initially designed by Roth et al. [24] The National Comprehensive Cancer Network (NCCN) has further developed the screening tool and added a problem list, which consists of 36 problems that are commonly experienced by cancer patients and grouped into five categories: practical, family, emotional, spiritual and physical [25]. The DT has been proven as an effective screening tool in patients with different types of cancer [22, 26, 27]. According to the NCCN guidelines, we defined a DT score of 5 or above indicating distress [25]. Regarding the five problem lists, any positive answer in either problem list was considered a positive result.

**PO-Bado** is a semi-directive instrument for assessing psychosocial difficulties in cancer patients constructed and validated in Germany [28–30]. It serves as an external assessment tool. In contrast to HADS and DT, the PO-Bado is completed by a psycho-oncologist in order to estimate the patient`s subjective experiences and psycho-social needs over the last three days. The different items are scored from 0-4. To detect patients with the need for psycho-oncological support, previously described stratification criteria are applied [29].

The HADS was used as a gold standard against which the other tests were compared. Independent from their screening results patients were asked, whether a psycho-oncological consultation was whished. The study was approved by the local ethic committees (study number 4087).

### Statistical analysis

Despite the prospective design of the study, all analyses were descriptive, therefore results were regarded as hypothesis generating only. A confirmatory set-up was not chosen. Statistical analyses were performed using the software IBM SPSS Statistics 24.0 (IBM, Armonk, New York, US). Data was described by standard statistics, using absolute and relative frequencies for categorical variables and median for continuous variables. Fisher’s exact test, chi-square test and univariate logistic regression modelling were used for categorical and continuous variables, respectively. All factors in the bivariate analyses were put into a multivariable logistic regression model. Odds ratios (OR) were obtained with corresponding 95% confidence intervals (CI). Patients with missing information about one variable were *only* excluded from the *corresponding* statistical analyses but not from the entire study. Sensibility and specificity as well as the number-needed to screen (NNS) were determined using cross-tables. In addition, receiver operator characteristic (ROC) curve analyses were conducted using HADS (HADS-A ≥ 11 or HADS-D ≥ 11) as the gold-standard against the other instruments. The area under the ROC curve (AUC= area under the curve) reflects the overall performance of the other test instruments in discriminating between patients with and without positive screening results in HADS. An AUC between 0.7 and 0.8 reflects fair discrimination results, between 0.8 and 0.9 a good and above 0.9 an excellent discrimination [31]. Pearson`s correlation coefficients were used to examine correlations. A probability value less than 0.05 was considered clinically relevant throughout the whole analyses. All reported p-values are two-sided.

Study protocol: M. Rapp, D. Wiewrodt

Conduct of study: K. Hoffmann and S. Schipman

Evaluation of data: M. Rapp, D. Wiewrodt, S. Schipman, K. Hoffmann, and R. Wiewrodt

Statistical analysis: S. Schipmann and R. Wiewrodt

Writing of manuscript: M. Rapp, D. Wiewrodt, S. Schipmann, and R. Wiewrodt

Editing and approval of manuscript: All authors.
